# A Deep Learning Based Approach for Localization and Recognition of Pakistani Vehicle License Plates

**DOI:** 10.3390/s21227696

**Published:** 2021-11-19

**Authors:** Umair Yousaf, Ahmad Khan, Hazrat Ali, Fiaz Gul Khan, Zia ur Rehman, Sajid Shah, Farman Ali, Sangheon Pack, Safdar Ali

**Affiliations:** 1 Department of Software Engineering, University of Sialkot, Sialkot 51040, Pakistan; umair.yousaf@uskt.edu.pk; 2 Department of Computer Science, COMSATS University Islamabad, Abbottabad Campus, Abbottabad 22044, Pakistan; fiazkhan@cuiatd.edu.pk (F.G.K.); ziarehman@cuiatd.edu.pk (Z.u.R.); 3 Department of Electrical and Computer Engineering, COMSATS University Islamabad, Abbottabad Campus, Abbottabad 22044, Pakistan; hazratali@cuiatd.edu.pk (H.A.); sajidshah@cuiatd.edu.pk (S.S.); 4 Department of Software, Sejong University, Seoul 05006, Korea; farmankanju@sejong.ac.kr; 5 School of Electrical Engineering, Korea University, Seoul 02841, Korea; 6 Department of Software Engineering, University of Lahore, Lahore 54000, Pakistan; safdar.ali@se.uol.edu.pk

**Keywords:** CNN, deep learning, license plate, localization, rectification, recognition, RNN

## Abstract

License plate localization is the process of finding the license plate area and drawing a bounding box around it, while recognition is the process of identifying the text within the bounding box. The current state-of-the-art license plate localization and recognition approaches require license plates of standard size, style, fonts, and colors. Unfortunately, in Pakistan, license plates are non-standard and vary in terms of the characteristics mentioned above. This paper presents a deep-learning-based approach to localize and recognize Pakistani license plates with non-uniform and non-standardized sizes, fonts, and styles. We developed a new Pakistani license plate dataset (PLPD) to train and evaluate the proposed model. We conducted extensive experiments to compare the accuracy of the proposed approach with existing techniques. The results show that the proposed method outperformed the other methods to localize and recognize non-standard license plates.

## 1. Introduction

Automatic license plate recognition has widespread applications in overcoming traffic violations, parking offenses, and better decision making for e-ticketing of vehicles [1]. A license plate is a crucial token issued by the state authority for vehicle identification and record keeping. Traffic wardens, tax collectors and other stockholders use license plates to monitor traffic and keep records accordingly. Modern traffic management systems rely heavily on automatic monitoring systems based on computer vision and machine learning techniques.

These typically require a license plate of standard size, color, font, style, and fixed location for automated localization and recognition. Unfortunately, the license plates currently used in Pakistan do not conform to standard features. In addition to a license plate, people also use different text to mention their profession, tribe, political affiliation, etc. This kind of text looks similar to license plates, which makes localization and recognition more difficult.

**Figure 1:**
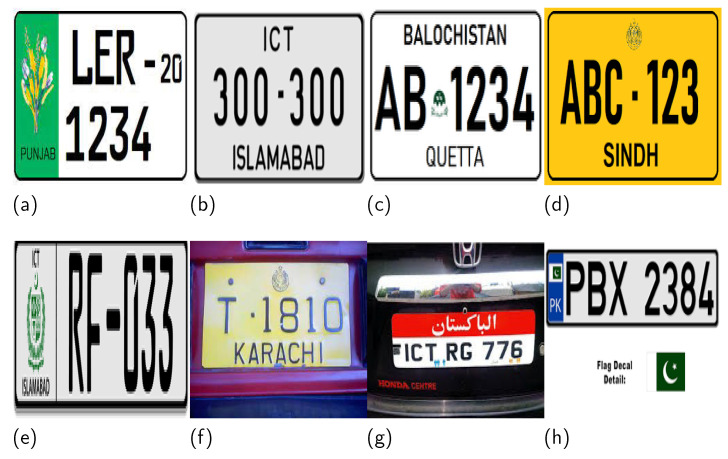
A few samples of Pakistani license plates. Each plate has a different font, color, and style. License plates in (**a**–**f**) are from different regions and (**g**,**h**) are customized plates which do not follow the the specifications.

For illustration, a few snaps of Pakistani license plates are shown in Figure 1. Note that the license plate in Figure 1a divides into two colors vertically: green and white. The green part contains a monogram and the text “PUNJAB”. Similarly, the text in the white part of the license plate consists of two lines in which the first line has different font sizes separated by a hyphen.

The license plates given in Figure 1b,c consist of three lines of text with gray and white backgrounds, respectively. The middle line in Figure 1b contains a dot that separates the digits, while the middle line in Figure 1c starts with two English alphabet letters, followed by a small monogram, and then four digits. If we closely observe the rest of Figure 1d–h, similar variations are visible. In such situations, the traditional license plate localization and recognition techniques fail to work [2].

The recent advancements in deep learning outperformed the traditional techniques in many fields of computer vision, such as object detection [3,4], segmentation [5,6,7,8], tracking [9,10], image understanding [11,12], image classification [13,14,15], medical diagnosis [16,17], and more [18,19]. A deep model requires a large amount of data for training (to extract out rich features and find the hidden nonlinear relationship among different entities). The successful implementation of deep learning in many fields of computer vision has inspired this research.

This paper presents a deep architecture to localize and recognize Pakistani license plates. Our model correctly localizes and recognizes the license plate when there are other texts and handles the color, illumination, size, style, and font variations. The main contributions of this work are:

- To train and test the model, a new Pakistani license plate dataset (PLPD) is developed.
- A deep end-to-end model is developed, which localizes, rectifies, and recognizes the uniform and non-uniform license plates.
- Detailed experiments are performed to compare the effectiveness of the proposed model with state-of-the-art methods.

The rest of the paper is organized as follows: the related work is discussed in Section 2, while Section 3, Section 4 and Section 5 present the proposed model, experimental details, and conclusion, respectively.

## 2. Related Work

License plate localization and recognition is considered to be a two-step process. Localization means finding a region in an image containing the license plate, while recognition means identifying the text written in it. Both the localization and recognition require feature extraction followed by classification. Based on feature extraction, the license plate localization and recognition methods can be divided into two classes, (1) hands-on feature engineering and (2) automated feature engineering. The hands-on feature engineering methods use core computer vision approaches to explicitly extract the features. On the other hand, automated feature engineering uses machine learning approaches to implicitly learn the features.

**Hands-on feature engineering methods:** Notable works on Pakistani license plate recognition based on hands-on feature engineering include Malik et al. [20] who used connected component analysis (CCA) to localize and recognize standard number plates of the Punjab province, which contain an inherent green region. They used the ratio between different color channels to locate the number plate area. For recognition purposes, template matching was used. Illumination changes, dust, and weather affect the color ratios and, thus, lead to imperfect detection. Similarly, the aspect ratio between green and white regions is exploited in HSV color space, to localize Punjab’s standard license plates [21].

In many works in this category [22,23,24], the Sobel edge detector is used to localize license plate and segment characters followed by template matching for recognition. The Sobel edge detector is very sensitive to noise because the first derivative leads to poor detection. Moreover, the Sobel detector is a scaled variant and fails to detect license plates of different sizes.

The histogram of vertical and horizontal edges was also employed for license plate localization [25]. A predefined threshold was used to analyze the license plate histogram. However, this approach lacks scale variations and distinguishes the license plate and other text phrases if they exist. Rasheed et al. [26] used Hough transformation (HT) to detect vertical and horizontal edges for localization. The Hough transform needs to manually specify a threshold to detect a line of a specific length. Moreover, this approach looks for rectangular regions and fails to distinguish a number plate and other rectangular regions.

Samra et al. [27] applied morphological operations followed by connected component analysis to get license plate proposals. The final license plate region was selected based on enclosed objects using a genetic algorithm (GA). In addition to derivatives and thresholding (prone to noise), it considers number plates as a sequence of fixed length and limits the application to standard license plates only.

In [28], the vertical edges were detected followed by AdaBoost to select the coarser level character-specific extremal regions (ER) for detection. Finally, the histogram of oriented gradient (HoG) was extracted, and the recognition was performed through hybrid discriminative restricted Boltzmann machines (HDRBMs). This approach considers a fixed aspect ratio between the height and width and restricts the application to Chinese standard number plates. Bhutta et al. [29] assumed a region as a license plate where characters lie on a straight line. They perform recognition using an SVM classifier.

**Automated feature engineering methods:** These methods use deep learning to implicitly learn the rich features. The deep-learning-based approaches for license plate recognition include [30,31], which generates license plate region proposals and performs the final selection using a CNN as a binary classifier. Similarly, the CNN is trained for the entire character sequence to detect and recognize Malaysian license plates [32].

The techniques above fail to distinguish license plates and other general alphanumeric text if they exist. Moreover, the fixed-width bounding box restricts the application to standard license plates. Zang et al. [33] applied a visual attention model to detect license plates containing blue and yellow regions. The final classification was performed with SVM. This approach is limited to certain types of number plates and weak to variations in illumination and scale.

Jain et al. [34] generated license plate proposals with a vertical Sobel filter and applied binary CNN for final verification. However, this failed to tackle the actual negative cases caused by Sobel due to noise. Laroca et al. [35] used two CNN models to detect vehicles and localize license plates, respectively. This method considers the number plate as a sequence of fixed length (of seven characters) and limits the application to standard license plates (of Brazil) only.

The work presented in [36] detects color edges followed by morphological analysis to extract the license plate. CCA and PA (projection analysis) with fixed height and width are used to segment characters, which is followed by recognition with CNN. Zhuang et al. [37] used semantic segmentation and counting refinement for recognition. This method works for fixed-length number plates and fails for varying lengths.

The methods discussed thus far have addressed license plate recognition for standard designs/sizes. However, the task of recognition for non-standard number plates remains a crucial gap. Hence, in this work, we propose a novel model to address the task of number plate recognition for varying sizes, fonts, styles, and designs.

## 3. Proposed Model

**Figure 2:**
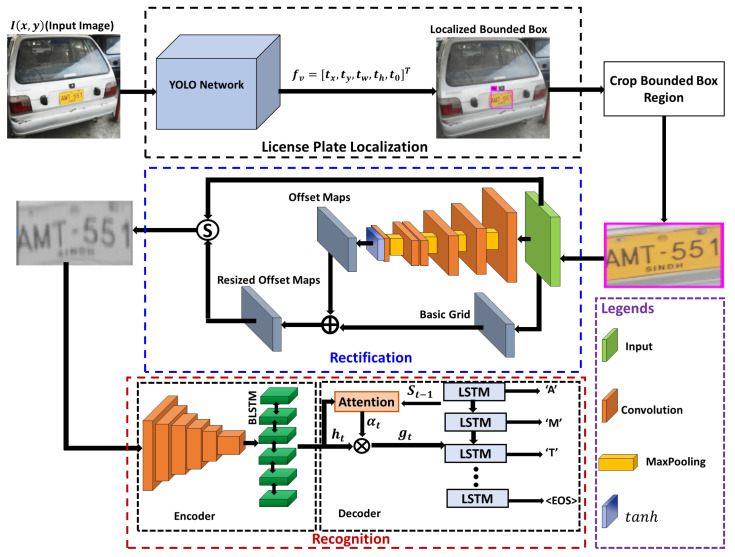
Block diagram of the system.

The proposed model consists of three modules: localization, rectification, and recognition. Figure 2 presents the block diagram of the proposed model. The localization module is responsible for finding the license plate area and drawing a bounding box around it. Typically, the license plates tilt or shear in a particular direction due to the vehicle motion or camera orientation. The curved and tilted nature of the text drops the recognition accuracy. For robust and accurate recognition, the text is rectified. Finally, the rectified image is passed to the recognition module, which converts the image text to an editable text. The following subsections discuss the proposed model in detail.

### 3.1. License Plate Localization

YOLO [38] is a fully convolutional neural network that contains layers with downsampling and upsampling and skips connections. It takes a RGB image $I\in {ℜ}^{416\times 416\times 3}$ and generates an output map $O\in {ℜ}^{13\times 13\times |f_{v}|}$, where $|f_{v}|$ is the length of the feature vector $f_{v}$ predicted by the network, and $f_{v}$ is given by [38]

(1) $$f_{v}={\left[t_{x},t_{y},t_{w},t_{h},t_{o}\right]}^{T}.$$

The given feature vector contains the bounding box information, where $\left(t_{x},t_{y}\right)$ is the midpoint of the bounding box relative to the cell, while $t_{w},t_{h}$, and $t_{o}$ represent the width, height, and type (class) of the bounding box, respectively. Note that the bounding boxes are divided into two classes: license plate and non-license plate. For license plates, $t_{o}=1$, and vice versa for non-license plates. The cell is offset by $\left(c_{x},c_{y}\right)$, and then the coordinates of the bounding box relative to the top left corner of the image are given by [38]:

(2)bx=11+e−tx+cx(3)by=11+e−ty+cy(4)bw=pwetw(5)bh=pheth

where $\left(b_{x},b_{y}\right)$, $b_{h}$, and $b_{w}$ represent the mid-point, height, and width of the resultant bounding box, respectively. $p_{h}$ and $p_{w}$ represent the height and width of the anchor box, respectively.

### 3.2. License Plate Rectification

As mentioned before, Pakistani license palates are non-standard and vary in color, font, style, and orientation. Moreover, license plate images may contain distortion, which further complicates the recognition. Thus, rectification is essential to increase recognition accuracy. The rectification may be performed with affine transformation, but it cannot handle variations in scale, rotation, and translation due to geometric constraints. Therefore, we used a trainable and constraint-free model; the multi-object rectified network (MORN) [39]. This uses deformable convolutional kernels to extract distinctive rich features that predict the offsets and rectify the text. Table 1 provides the model summary [39].

MORN applies ReLU to the batch normalized output of each convolutional layer except the last. It splits the image into different parts and then estimates the offset of each part. The tanh(·) is used to return the offset values in the range of (−1,1), representing the relative position to the original position. The relative offset map is resized to the size of the input using bilinear interpolation.

A basic grid in the range [−1,1] is generated from the input image to remember the positions of the original pixels. Note that the coordinates of the top-left and bottom-right pixels are denoted by (−1,−1) and (1,1), respectively. Let α represents the relative offset map, and β represents the basic grid then the new offset map $\bar{\alpha }$ is given by:

(6) $$\bar{\alpha }\left(i,j,c\right)=\alpha \left(i,j,c\right)+\beta \left(i,j,c\right);c=1,2$$

where *c* represents the channel and (i,j) represent the respective coordinates. The offset map $\bar{\alpha }\left(i,j,c\right)$ is mapped to the size of image such that i∈[0,W] and j∈[0,H], where *H* and *W* represent the height and width of an image *I*. Thus, the pixel value at (i,j) of the rectified image $\bar{I}$ is given by:

(7) $$\bar{I}\left(i,j\right)=I\left(\bar{\alpha }\left(1,i,j\right),\bar{\alpha }\left(2,i,j\right)\right).$$

Note that $\bar{\alpha }\left(1,i,j\right)$ and $\bar{\alpha }\left(2,i,j\right)$ sample the first and second coordinates from the first and second channels of the offset map.

### 3.3. License Plate Recognition

Recognition is the process to convert an image-text to editable text. For recognition, we used an attention sequence-to-sequence network [40], which takes the rectified image as an input and predicts the license plate character sequence. The detailed architecture of the recognition network is given in Table 2. The presented model consists of an encoder and a decoder.

The encoder module uses a stack of convolutional layers (ConvNet) that extract rich and discriminative features. First, the ConvNet scans the rectified image and generates a feature map of height one using scaling. Next, the feature map is split along the row axis and transformed into a feature vector *v*. Next, a multi-layer bidirectional long short-term memory (BLSTM) network is used to analyze the feature vector. The BLSTM bidirectionally captures the long-term dependencies in feature vector *v* and generates a new feature vector $v_{n}$ of the same length, see [40] for details.

The decoder is based on an attention sequence-to-sequence bidirectional model. It processes the input sequence vector (decoder output) $v_{n}$, from left-to-right and right-to-left and generates two output sequences $S_{lr}$ and $S_{rl}$, respectively. The output results $S_{lr}$ and $S_{rl}$ are merged on the basis of the height recognition score.

## 4. Experimental Details

### 4.1. Datasets

There is no publicly available dataset for Pakistani license plates. Therefore, one of our main contributions is in creating our dataset, called the Pakistani License Plate Dataset (PLPD). This dataset contains 6000 images of license plates that vary in style, font, color, illumination, and view angles. Moreover, we generated ground truth (annotations) for each image. The annotation consists of the bounding box information (height, width, and midpoint) and the license plate text. Finally, the dataset is split into training and validation sets with a ratio of 80% and 20%, respectively.

The performance of the proposed approach is also compared with other methods using two publicly available datasets: artificial Mercosur license plates (AMLP) [41] and the Roboflow license plate dataset (RLPD) [42]. AMLP [41] and the RLPD [42] contain 3839 and 350 images with ground truth annotations, respectively.

### 4.2. Performance Measures

The intersection over union (IoU) is used to evaluate the localization results. This estimates that how much the predicted bounding box matches the ground truth and is given by:

(8) $$\mathrm{IoU}=\frac{\mathrm{Area}\;\mathrm{of}\;\mathrm{Overlap}}{\mathrm{Area}\;\mathrm{of}\;\mathrm{Union}}$$

The IoU returns a score in the range of [0,1]; a higher score indicates good localization and vice versa. Moreover, the recall, precision, F1 score, and accuracy are used to evaluate the recognition results.

### 4.3. Results

**Figure 3:**
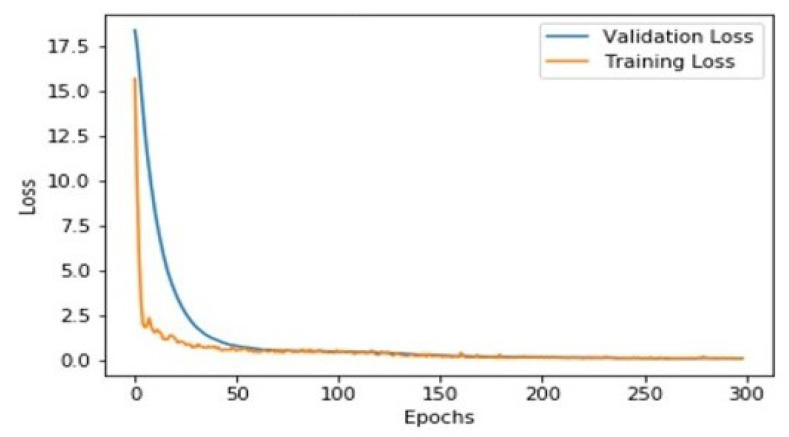
Training loss and validation losses.

**Figure 4:**
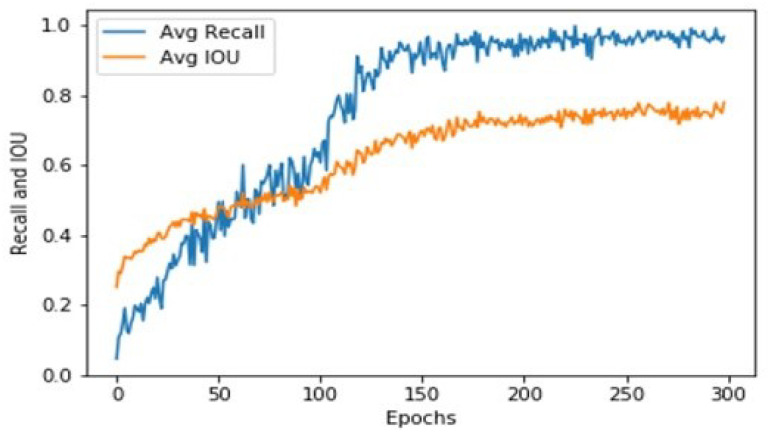
IOU and recall.

The model is trained for 300 epochs. The training and validation losses for localization are shown in Figure 3. In the first iteration, the training loss was 18, and the validation loss was 15.3. Both the losses decreased with successive iterations and converged at 0.1127 and epoch 300. Similarly, Figure 4 shows the average intersection over union (IoU) and average recall scores for different iterations during training. Initially, the IoU and recall started at 0.2227 and 0.1 and achieved final scores of 0.8 and 0.9.

**Figure 5:**
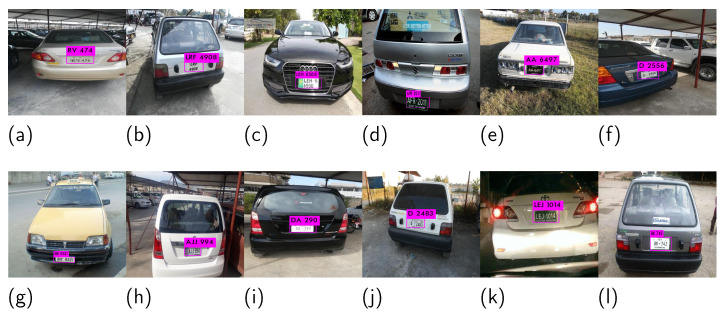
Localization and recognition results of the proposed approach on the PLPD dataset images. (**a**,**b**,**g**) are non-standard customized plates while the remaining subfigures show standared plates with different regional designs. In (**g**,**d**) the license plate is recognised but other text is ignored.

Figure 5 shows the localization and recognition results of the proposed approach for different images taken from the PLPD. The given images vary in terms of style, font, and orientation. The license plate in Figure 5a contains an unknown font where the digits are preceded by a space and then two English alphabet characters. In Figure 5b,c, the license plates consist of two lines with variations in terms of hyphen and monogram. Similarly, the license plates in Figure 5d–l contain one line but vary in terms of orientation, number alphabets, the number of digits, and their separating symbols (-, *).

The license plates in Figure 5d,k,l contain extra text, such as “GOVT OF SINDH”, “GOVT OF PAKISTAN”, and “ICT Islamabad”, respectively. In Figure 5l, the extra phrases “CHAKWAL” and “SUZUKI CHAKWAL” can be seen, which look like license plates. However, the proposed pipeline perfectly localized and recognized the license plates irrespective of the mentioned variations.

**Figure 6:**
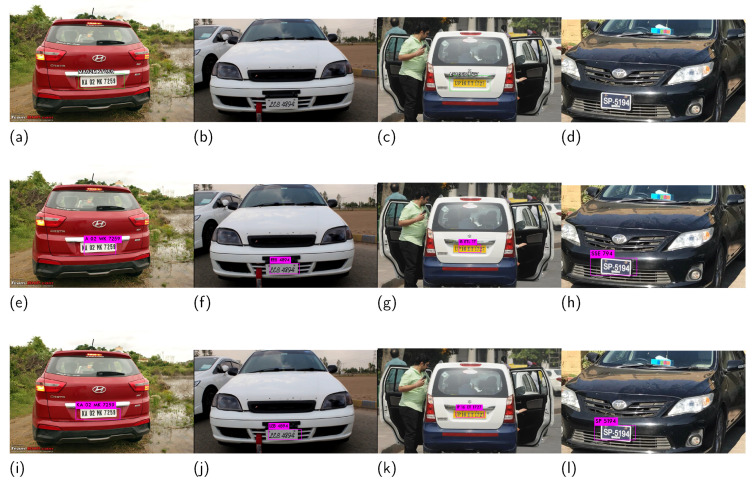
A qualitative comparison with DALPR [34] and KLPR [43] (**a**–**d**) show the results of DALPR, (**e**–**h**) of KLPR, and (**i**–**l**) of the proposed method, respectively.

The results of the proposed approach were compared with two recent approaches: deep automatic license plate recognition system (DALPR) [34] and Korean license plate recognition system using combined neural networks (KLPR) [43]. The methods mentioned above use deep learning to localize and recognize Indian and Korean license plates. Figure 6 shows the qualitative comparison of the proposed method with DALPR [34] and KLPR [43] for images. Figure 6a–d, presents the results of DALPR [34], while images in Figure 6e–l depict the results of KLPR [43] and the proposed method, respectively.

The qualitative results show that the proposed method was able to localize the license plate and recognize the text accurately. Our method consistently outperformed the two other methods. Moreover, DALPR [34] failed to localize the license plates in certain cases, such as Figure 6b,d.

**Figure 7:**
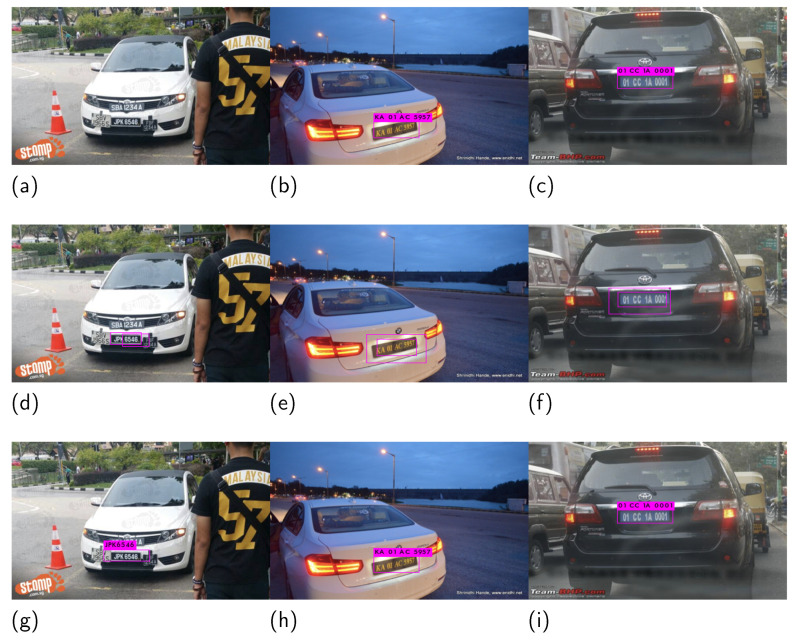
Images taken under different conditions. (**a**–**c**) present the results of DALPR [34], (**d**–**f**) show the results of KLPR [43] while (**g**–**i**) are the results of our proposed method, respectively.

Figure 7 presents the results under different conditions, such as complex situations, evening, and fog. The first row in Figure 7 depicts a complex situation where the front of the car contains a license plate “JPK 6546”, which is surrounded by three other number plate-like tokens, including “SBA 1234A” (top), “SBV 966S” (left), and “FBF 1234A” (right). In this situation, DALPR [34] failed to detect the license plate while KLPR [43] drew two bounding boxes but failed to recognize even a single letter.

Note that the proposed method perfectly detected and recognized the license plate. Similarly, the second and third columns of Figure 7 present the results under low light, such as evening (second column) and fog (third column) conditions. In both situations, KLPR detected the license plate with double bounding boxes and failed in the case of recognition. On the other hand, both DALPR and the proposed method worked fine in the tested low lighting situations.

Table 3 compare the localization results in term of the intersection over union (IoU). Table 3 shows that the proposed approach outperformed the other methods and achieved the best scores for the IoU. To evaluate the recognition performance, the proposed method was compared with DALPR [34] and KLPR [43] using three datasets: the Pakistani license plate dataset (PLPD), artificial Mercosur license plates (AMLP) dataset [41], and Roboflow license plate dataset (RLPD) [42].

Table 4, Table 5 and Table 6 present the quantitative analysis of the recognition results in term of the accuracy, recall, precision, and F1 score. It is clear from the mentioned tables that the proposed method outperformed the other methods and achieved the best score. Note that the DALPR [34] method failed in the case of the AMLP [41] and RLPD [42] datasets because it is designed to handle license plates of fixed length, standard color, and standard format. The proposed method is more general and robust to variations in license plate styles and orientation.

## 5. Conclusions

In this paper, we presented a deep-learning-based approach for the localization and recognition of Pakistani license plates, which vary in font and style. The proposed method comprises three steps: localization, rectification, and recognition. The localization detects the license plate in the image and extracts the region of interest. In some cases, the view angle between the camera and car affects the license plate’s orientation and leads to shearing effects. The mentioned shearing effects make recognition difficult due to volumetric strain and changes in style. To handle the shearing effects and uniformly align the text, rectification is used. Finally, recognition is performed.

The proposed pipeline was evaluated on a newly developed dataset of Pakistani license plates, i.e., the PLPD, which contains 6000 images covering various Pakistani license plates. Extensive experiments were performed to compare the performance of the proposed approach with state-of-the-art deep learning methods. The results show that the proposed approach performed better compared to other methods. In the future, this work may be extended to localize and recognize license plates written in other languages, such as Urdu and Pashto.

## Figures and Tables

**Table 1:** The MORN architecture [39]: *k*, *s*, *p*, *conv*, and *MaxPool* represent the kernel, stride, padding, convolution layer, and max pooling layer, respectively.

Layer Type	Hyper-Parameters	Size
Input	–	1×32×100
MaxPool	k2×2,s2×2	1×16×50
Conv	64,k3×3,s1×1,p1×1	64×16×50
MaxPool	k2×2,s2×2	64×8×25
Conv	128,k3×3,s1×1,p1×1	128×8×25
MaxPool	k2×2,s2×2	128×4×12
Conv	64,k3×3,s1×1,p1×1	64×4×12
Conv	16,k3×3,s1×1,p1×1	16×4×12
Conv	2,k3×3,s1×1,p1×1	2×4×12
MaxPool	k2×2,s2×2	2×3×11
tanh	–	2×3×11
Resize	–	2×32×100

**Table 2:** The recognition network [40] architecture: where each block is residual and * represents the dynamic output length.

	Layers	Out Size	Configuration
Encoder	Block 0	32×100	3×3conv,s1×1
	Block 1	16×50	$\left\{\begin{array}{c} 1\times 1\;\mathrm{conv},32\\ 3\times 3\;\mathrm{conv},32 \end{array}\right\}\times 3,\;s\;2\times 2$
	Block 2	8×25	$\left\{\begin{array}{c} 1\times 1\;\mathrm{conv},64\\ 3\times 3\;\mathrm{conv},64 \end{array}\right\}\times 4,\;s\;2\times 2$
	Block 3	4×25	$\left\{\begin{array}{c} 1\times 1\;\mathrm{conv},128\\ 3\times 3\;\mathrm{conv},128 \end{array}\right\}\times 6,\;s\;2\times 1$
	Block 4	2×25	$\left\{\begin{array}{c} 1\times 1\;\mathrm{conv},256\\ 3\times 3\;\mathrm{conv},256 \end{array}\right\}\times 6,s\;s\;\times 1$
	Block 5	1×25	$\left\{\begin{array}{c} 1\times 1\;\mathrm{conv},512\\ 3\times 3\;\mathrm{conv},512 \end{array}\right\}\times 3,\;s\;2\times 1$
	BiLSTM l	25	256 hidden units
	BiLSTM 2	25	256 hidden units
Decoder	Att. LSTM	*	256 attention units
			256 attention units
	Att. LSTM	*	256 attention units
			256 attention units

**Table 3:** Comparison of the localization results.

Model	IOU
DALPR [34]	0.60
KLPR [43]	0.72
Proposed method	0.89

**Table 4:** Recognition results on the Pakistani license plate dataset (PLPD).

Model	Accuracy	Recall	Precision	F1 Score
DALPR [34]	0.20	0.37	0.80	0.50
KLPR [43]	0.53	0.70	0.87	0.77
Proposed method	0.82	0.99	0.94	0.96

**Table 5:** Recognition results on the artificial Mercosur license plates (AMLP) [41].

Model	Accuracy	Recall	Precision	F1 Score
DALPR [34]	0.00	0.00	0.00	0.00
KLPR [43]	0.70	0.82	0.79	0.80
Proposed method	0.87	0.96	0.93	0.94

**Table 6:** Recognition results on the Roboflow license plate dataset (RLPD) [42].

Model	Accuracy	Recall	Precision	F1 Score
DALPR [34]	0.00	0.00	0.00	0.00
KLPR [43]	0.79	0.75	0.72	0.73
Proposed method	0.89	0.94	0.90	0.91
